# Complex structural variants in Mendelian disorders: identification and breakpoint resolution using short- and long-read genome sequencing

**DOI:** 10.1186/s13073-018-0606-6

**Published:** 2018-12-07

**Authors:** Alba Sanchis-Juan, Jonathan Stephens, Courtney E. French, Nicholas Gleadall, Karyn Mégy, Christopher Penkett, Olga Shamardina, Kathleen Stirrups, Isabelle Delon, Eleanor Dewhurst, Helen Dolling, Marie Erwood, Detelina Grozeva, Luca Stefanucci, Gavin Arno, Andrew R. Webster, Trevor Cole, Topun Austin, Ricardo Garcia Branco, Willem H. Ouwehand, F. Lucy Raymond, Keren J. Carss

**Affiliations:** 1 Department of Haematology, University of Cambridge, NHS Blood and Transplant Centre, Cambridge, CB2 0PT UK; 2 NIHR BioResource, Cambridge University Hospitals NHS Foundation Trust, Cambridge Biomedical Campus, Cambridge, CB2 0QQ UK; 3 Department of Paediatrics, University of Cambridge, Cambridge, CB2 0QQ UK; 4 Cambridge University Hospitals NHS Foundation Trust, Cambridge, CB2 0QQ UK; 5 Department of Medical Genetics, Cambridge Institute for Medical Research, University of Cambridge, Cambridge, CB2 0XY UK; 6 National Health Service Blood and Transplant (NHSBT), Cambridge Biomedical Campus, Cambridge, CB2 0PT UK; 7 BHF Centre of Excellence, Division of Cardiovascular Medicine, Addenbrooke’s Hospital, Cambridge Biomedical Campus, Cambridge, CB2 0QQ UK; 8 UCL Institute of Ophthalmology, University College London, London, EC1V 9EL UK; 9 Moorfields Eye Hospital NHS Trust, London, EC1V 2PD UK; 10 West Midlands Genomic Medicine Centre, University Hospitals Birmingham, Birmingham, UK

**Keywords:** Genome sequencing, Next-generation sequencing, Complex structural variant, Nanopore, *ARID1B*, *HNRNPU*, *CEP78*, *CDKL5*

## Abstract

**Background:**

Studies have shown that complex structural variants (cxSVs) contribute to human genomic variation and can cause Mendelian disease. We aimed to identify cxSVs relevant to Mendelian disease using short-read whole-genome sequencing (WGS), resolve the precise variant configuration and investigate possible mechanisms of cxSV formation.

**Methods:**

We performed short-read WGS and analysis of breakpoint junctions to identify cxSVs in a cohort of 1324 undiagnosed rare disease patients. Long-read WGS and gene expression analysis were used to resolve one case.

**Results:**

We identified three pathogenic cxSVs: a de novo duplication-inversion-inversion-deletion affecting *ARID1B*, a de novo deletion-inversion-duplication affecting *HNRNPU* and a homozygous deletion-inversion-deletion affecting *CEP78*. Additionally, a de novo duplication-inversion-duplication overlapping *CDKL5* was resolved by long-read WGS demonstrating the presence of both a disrupted and an intact copy of *CDKL5* on the same allele, and gene expression analysis showed both parental alleles of *CDKL5* were expressed. Breakpoint analysis in all the cxSVs revealed both microhomology and longer repetitive elements.

**Conclusions:**

Our results corroborate that cxSVs cause Mendelian disease, and we recommend their consideration during clinical investigations. We show that resolution of breakpoints can be critical to interpret pathogenicity and present evidence of replication-based mechanisms in cxSV formation.

**Electronic supplementary material:**

The online version of this article (10.1186/s13073-018-0606-6) contains supplementary material, which is available to authorized users.

## Background

Structural variants (SVs) are a major source of variation in the human genome and collectively account for more differences between individuals than single nucleotide variants (SNVs) [1, 2]. SVs are categorised as canonical or complex [3]. The canonical forms can be balanced or unbalanced and comprise inversions, insertions, translocations, deletions and duplications. More complex rearrangements are typically composed of three or more breakpoint junctions and cannot be characterised as a single canonical SV type. These are known as non-canonical or complex SVs (cxSVs) [3, 4].

Several previous studies have reported clinically relevant cxSVs in individuals with Mendelian disorders. For example, a duplication-triplication-inversion-duplication was found at the *MECP2* and *PLP1* loci in individuals with MECP2 duplication syndrome or Lubs syndrome (MIM: 300260) and Pelizaeus-Merzbacher disease (MIM: 312080) [5, 6], and a duplication-inversion-terminal deletion of chromosome 13 was present in foetuses with 13q deletion syndrome [7], among others [8–10]. Recently, pathogenic cxSVs associated with autism spectrum disorder and neuropsychiatric disorders have also been reported [11, 12]. Whole-genome sequencing (WGS) studies have shown that cxSVs are considerably more abundant and diverse than had been previously appreciated, representing an estimated 2% of the SVs in the human genome, and each human genome contains on average 14 cxSVs [11]. The presence of multiple types of cxSVs has also been independently observed in several other studies [5, 12–14]. Extreme cases of cxSVs, such as chromothripsis, have also been identified in both cancer cells and the germline and involve hundreds of rearrangements often concerning more than one chromosome [11, 15].

Nevertheless, cxSVs are not typically considered during genomic analysis, largely due to technical challenges of identification. Complex SVs have been reported in projects such as the 1000 Genomes, but these primarily focused on the canonical types [1, 16, 17]. With the rapid expansion of high-throughput sequencing technologies including long-read WGS, genome-wide characterisation of SVs with high precision has been achieved [1], facilitating the study of more complex forms of SVs.

Therefore, in the present study, we sought to investigate the role of cxSVs in Mendelian disease by first identifying potentially clinically relevant cxSVs in a subset of the NIHR BioResource project using short-read WGS, second, resolving the variant configuration to base pair level resolution and, third, investigating possible mechanisms of cxSV formation by breakpoint analysis.

## Methods

### Cohort description

This cohort comprises 1324 individuals from the NIHR BioResource research study, which performs WGS of individuals with undiagnosed rare disorders. It is composed of three different subprojects: 725 were in the Inherited Retinal Disorders (IRD) project, 472 were in the Neurological and Developmental Disorders (NDD) project and 127 were in the Next Generation Children (NGC) project, which performs diagnostic trio WGS of individuals from Neonatal and Paediatric Intensive Care Units.

### Short-read WGS and variant identification

We performed short-read WGS and excluded the possibility of pathogenic SNVs or indels, as part of the NIHR BioResource project as previously described [18]. For the NDD and IRD subprojects we restricted SNVs and indel analysis to known disease-associated genes, which we assembled from sources including OMIM, RetNet and literature searches, then curated to ensure they comply with previously described criteria [19]. The lists comprise 1423 genes (NDD) and 248 genes (IRD). For NGC participants, trio analysis focused on de novo and rare biallelic variant discovery unrestricted by a gene list.

The first stage of cxSV identification was calling and filtering canonical SVs. These initial calls comprise simple canonical SVs, and those which were potentially individual segments of cxSVs were then identified by clustering. These canonical SVs were called by Canvas [20], which identifies copy number gains and losses based on read depth, and Manta [21], which calls translocations, deletions, tandem duplications, insertions and inversions, and is based on both paired read fragment spanning and split read evidence. SVs were initially filtered to keep only those that pass standard Illumina quality filters, do not overlap previously reported CNVs in healthy cohorts [22] and are rare (minor allele frequency < 0.01) in the whole NIHR BioResource study (*n* = 9453) Schematic of the workflow can be found in Additional file 1: Figure S1.

### Identification of potentially clinically relevant cxSVs

To identify potentially clinically relevant cxSVs we first identified putative cxSVs in the 1324 individuals by clustering canonical SV calls from Canvas and Manta using Bedtools cluster allowing a maximum distance between calls of 1 Kb [23]. We then categorised the putative cxSVs into different subtypes previously described [11].

Next, as the scope of this study was limited to the identification of potentially clinically relevant cxSVs, we performed strict post-processing on the list of putative cxSVs. We excluded any for which visual inspection of the reads in IGV suggested the cxSV was not real but an artefact of a region of low sequencing quality and any in a gene that was not consistent with both the expected genotype and phenotype of the patient. We also filtered out retrotransposons, which are miscalled as multiple clustered intronic deletions, and dispersed duplications, which are frequently miscalled as an overlapping deletion and tandem duplication [24].

### Validation

Sanger sequencing of the PCR product of the breakpoints was performed using standard protocols. Copy number variable segments of cxSVs and regions of homozygosity were confirmed using Illumina SNP genotyping array as previously described [18], and/or CytoScan® 750 K Cytogenetics Solution microarray (Affymetrix).

To resolve the configuration of the cxSV in participant 4 (P4), we performed long-read WGS with Oxford Nanopore Technologies (ONT). The sample was prepared using the 1D ligation library prep kit (SQK-LSK108), and genomic libraries were sequenced on R9 flowcell. Read sequences were extracted from base-called FAST5 files by albacore (version 2.0.2) to generate FASTQ files and then aligned against the GRCh37/hg19 human reference genome using NGMLR (version 0.2.6) [25] and LAST (version 912) [26], in order to compare results. Analysis was performed using default parameters, and for LAST, we used first last-train function to optimise alignment scoring. Variant calling was performed with Sniffles [25] and NanoSV [27], respectively.

RNA gene expression analysis of *CDKL5* was as also performed on P4 and both parents. RNA was extracted from blood using the PAXgene Blood RNA Kit (QIAGEN) and retro-transcribed using the High Capacity cDNA Reverse Transcription Kit (Thermo Fisher Scientific). We performed PCR amplification and Sanger sequencing of the informative SNP rs35478150 (X:g.18638082A>C).

### Breakpoint flanking sequence analysis

We analysed the sequence flanking each confirmed breakpoint (+/− 150 bps) and manually identified microhomology. The percentage of repetitive sequence was then calculated using RepeatMasker version open-4.0.7 (http://www.repeatmasker.org). In order to identify de novo SNVs and indels at the breakpoint junctions, SNV/indel calling and de novo filtering was performed with Platypus (http://github.com/andyrimmer/Platypus) for those participants for which parental WGS was available (P1 and P4).

## Results

### Potentially clinically relevant cxSVs in four individuals with Mendelian disease

We identified four individuals with potentially clinically relevant cxSVs. Participant 1 (P1) presents a de novo duplication-inversion-inversion-deletion encompassing *ARID1B* (MIM: 135900) that causes Coffin-Siris syndrome (CSS [MIM: 135900]). This individual was a 4-month-old female who was born prematurely and presented with characteristic features of CSS as a neonate. CSS is a multiple malformation syndrome characterised by intellectual disability, severe speech impairment, coarse facial features, microcephaly, developmental delay and hypoplastic nails of the fifth digits [28].

**Fig. 1:**
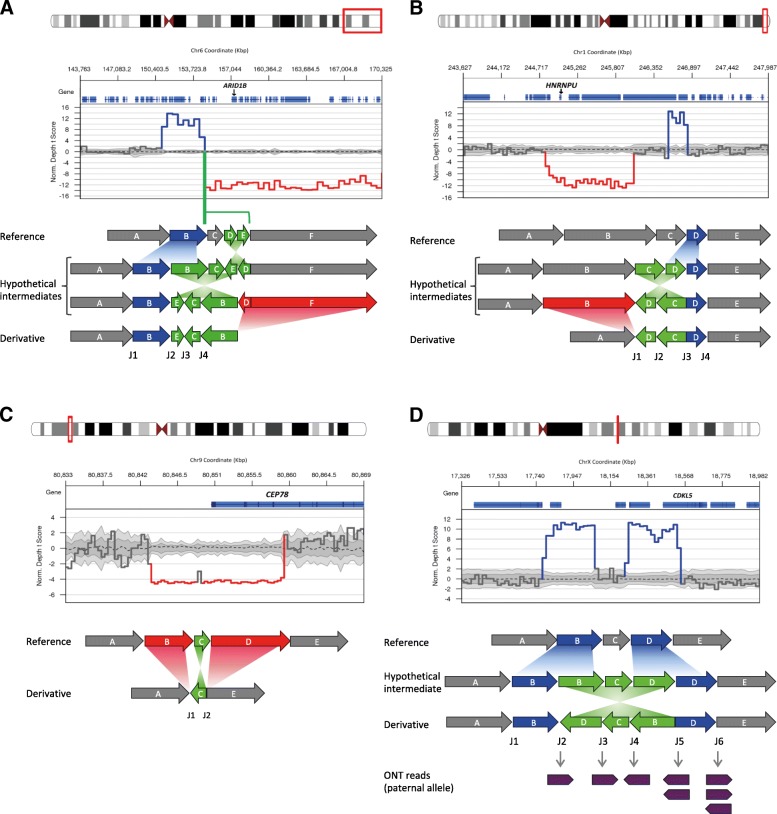
Four complex structural variants identified by genome sequencing. Plots show changes in coverage of short-read WGS (normalised depth *t* score using CNView, *n* = 250) [48]. Schematic models show the possible sequences of mutational events leading to the formation of the confirmed cxSVs, including putative intermediate derivative chromosomes where relevant. Sizes of fragments are approximately to scale where possible. An extended version of this figure showing breakpoint junction sequences is provided in Additional file 1: Figure S5, and alternative models for P4 are provided in Additional file 1: Figure S2. **a** A duplication-inversion-inversion-deletion causes Coffin-Siris syndrome in P1. **b** A deletion-inversion-duplication causes intellectual disability and seizures in P2. **c** A deletion-inversion-deletion causes cone-rod dystrophy in P3. **d** A duplication-inversion-duplication overlaps with *CDKL5* in P4, who had neonatal hypoxic-ischaemic encephalopathy. Oxford Nanopore Technology (ONT) long-read WGS confirms the presence of a disrupted (J2) and intact (J6) copy of the gene. Only paternally inherited reads overlapping the junction breakpoints are shown

A large cxSV was identified on chromosome 6, comprising a 3.3 Mb duplication, two inversions of 4.9 Kb and 3.3 Mb, and a 16.3 Mb deletion (Fig. 1a; Table 1). A total of 87 protein-coding genes were within the structural variant boundaries (Additional file 2: Table S1), of which 21 have been previously described as disease-associated in OMIM. The 16.3 Mb deletion contains 72 genes, of which only 6 have been reported as associated with autosomal dominant disease or constrained for loss-of-function (LOF) variation in ExAC [29] (Additional file 2: Table S1). Of these 6, only *ARID1B* has been previously reported as disease-associated with a LOF mechanism. Haploinsufficiency of *ARID1B* causes CSS, consistent with the phenotype of P1. We also looked at the 10 autosomal recessive genes within the deletion and did not find a second likely pathogenic variant in any. No disease-associated gene that was present within the duplicated region had been reported to be triplosensitive. Furthermore, the first inversion and the 3′ breakpoint of the second inversion were within *CNKSR3* (MIM: 617476). However, *CNKSR3* has not previously been associated with disease and is not constrained for LOF variation in ExAC; thus, the effect of this inversion on the phenotype remains unknown.

**Table 1:** Characteristics of participants, complex structural variants, and individual rearrangement events

Details of participant			Details of whole cxSV						Details of individual rearrangement events					
Participant	Phenotype	Sex	cxSV type	Nomenclature	Inheritance	Pathogenicity (implicated gene)	GT	Chr	Start	End	SV	Included segments^b^	Size	Confirmation method
P1	Coffin-Siris syndrome; Atrial septal defect; Cleft soft palate	F	dupINVinvDEL	46,XX,der(6)(q25.1,q28)dn seq[GRCh37/hg19]der(6)(6pter->6q25.2(+)(154768570):: q25.2(+)(154778901),q25.2(+)154778992:: q25.2(-)(154774047),q25.2 (-)(154768570):: q25.2(-)(154768570),q25.1(-)(~151443182-151443482))dn	De novo	Pathogenic (*ARID1B*)	Het	6	151443332^a^	154768570	Dup	B	3.3 Mb	Microarray
									151443332	154778901	Inv	B-C-E	3.3 Mb	Sanger
									154774047	154778992	Inv	D-E	4.9 kb	Sanger
									154778901	171115067	Del	D-F	16.3 Mb	Sanger and Microarray
P2	Tonic-clonic seizures; Intellectual disability; Learning difficulties	M	delINVdup	46,XX,der(1)(q44,q44). seq[GRCh37/hg19]der(1) (1pter->1q44(+)(244867200):: TCGCC{5}:: q44(-)(246816211),q44(-)(246064238}):: GGG...TAG{48}:: q44(+)(246569871)->1qter)dn	De novo	Pathogenic (*HNRNPU*)	Het	1	244867200	246064238	Del	B	1.2 Mb	Sanger and Microarray
									246064238	246816211	Inv	C-D	505 kb	Sanger
									246569871	246816211	Dup	D	246 kb	Sanger and Microarray
P3	Cone-rod dystrophy; Sensorineural hearing loss	M	delINVdel	46,XX,der(9)(q21.2,q21.2) seq[GRCh37/hg19]der(9) (9pter->9q21.2(+)(80843698):: q21.2(-)(80849760}),q21.2(-)(80849462}):: q21.2(+)(80859678}->9qter)	Not available	Pathogenic (*CEP78*)	Hom	9	80843698	80849462	Del	B	5.7 kb	Sanger and Microarray
									80849462	80849760	Inv	C	298 bp	Sanger
									80849760	80859678	Del	D	9.9 kb	Sanger and Microarray
P4	HIE Grade 2, birth asphyxia; Fetal distress; Intrauterine hypoxia	F	dupINVdup	46,XX,der(X)(p22.13,p22.13)dn seq[GRCh37/hg19]der(X) (Xpter->Xp22.13(+)(18074005):: GCA…CAC{100}:: p22.13(-)(18532312),p22.13(-)(17793009):: p22.13(+)(18248955)->Xqter)dn	De novo	VUS (*CDKL5*)	Het	X	17793009	18074005	Dup	B	280 kb	Sanger and Microarray
									17793009	18532312	Inv	B-C-D	458 kb	Sanger and ONT
									18248955	18532312	Dup	D	283 kb	Microarray

cxSVs are described using Next-Gen Cytogenetic Nomenclature [49]. For details of all affected genes, see Additional file 2: Table S1

*cxSV* complex structural variant, *GT* genotype, *Chr* chromosome, *SV* structural variant

^a^Not confirmed by Sanger sequencing; coordinate obtained from direct observation of WGS data in IGV

^b^Refers to genomic segments as shown in Fig. 1

Although the LOF of *ARID1B* likely explains the phenotype of this individual, it is possible that other genes affected by the cxSV might contribute to the phenotype. Examination of the parental origin of the hemizygous variants in the deleted region confirmed that the cxSV occurred on the paternal chromosome, consistent with previously reported observations that ~ 80% of de novo mutations are of paternal origin [30].

Participant 2 (P2) has a de novo deletion-inversion-duplication encompassing *HNRNPU* (MIM: 602869). This individual is a 22-year-old male who presented at term with hypotonia. All his early developmental milestones were delayed, and he presented with tonic-clonic seizures at 9 months. His seizure disorder has been managed by medication but has continued episodically into adulthood. He also has significant intellectual disability, autism, and limited speech and language, and MRI showed partial agenesis of the corpus callosum and enlarged ventricles.

We identified a cxSV on chromosome 1, formed by a 1.2 Mb deletion and a 246 Kb duplication flanking an inversion of 505 Kb (Fig. 1b; Table 1). This variant encompassed eight genes (Additional file 2: Table S1), of which two were previously associated with disease: *COX20* (MIM: 614698) and *HNRNPU*, both within the deletion boundaries. Haploinsufficiency of *COX20* was not deemed likely to be pathogenic as variants in this gene have an autosomal recessive mode of inheritance and result in a mitochondrial complex IV deficiency (MIM: 220110), which is not consistent with the individual’s phenotype, and no second rare variant was identified. However, *HNRNPU* is a highly constrained gene for LOF variants, in which haploinsufficiency causes early infantile epileptic encephalopathy (EIEE [MIM: 617391]). Microdeletions of *HNRNPU* have been described in individuals with intellectual disability and other clinical features, such as seizures, corpus callosum abnormalities and microcephaly [31].

Participant 3 (P3), a 66-year-old male, presented with a cone-rod dystrophy and hearing loss due to a homozygous deletion-inversion-deletion overlapping *CEP78* (MIM: 617110). Onset was in his fifth decade with central vision loss, photophobia and nystagmus accompanied by progressive hearing impairment, following a severe influenza-like viral infection. Two homozygous deletions in chromosome 9 of nearly 6 and 10 Kb were found flanking an inversion of 298 bp (Fig. 1c; Table 1). The second deletion intersects with the first 5 exons of *CEP78*. Biallelic LOF variants in this gene have been previously shown to cone-rod dystrophy and hearing loss (MIM: 617236) [32]. Although we could not perform segregation analysis due to lack of parental DNA, it was observed to be within a copy number neutral region of homozygosity that comprised approximately Chr9:70984372-86933884.

Participant 4 (P4) presents a duplication-inversion-duplication overlapping *CDKL5* (MIM: 300203) on chromosome X. This individual was a female term (41 + 1) neonate who presented with foetal bradycardia. She was diagnosed with hypoxic-ischemic encephalopathy grade 2, intrauterine hypoxia, and perinatal asphyxia, with poor cord gases. Hypothermia was induced after birth for 72 h to reduce brain injury. WGS revealed a de novo duplication-inversion-duplication, with the respective sizes of 280 Kb, 458 Kb and 283 Kb (Fig. 1d; Table 1). The inversion 3′ breakpoint is in intron 3 of 20 of *CDKL5* (NM_003159). Heterozygous rare variants in X-linked *CDKL5* in females cause EIEE, severe intellectual disability and Rett-like features (MIM: 300672). There are three other genes within the boundaries of this cxSV, none of them disease-associated in OMIM (Additional file 2: Table S1).

### Accurate resolution of variant configuration is necessary for interpretation of pathogenicity

For each of the four cases, we validated each breakpoint in order to resolve the variant configuration to base pair level resolution. For P1, P2 and P3, all novel junctions were confirmed by Sanger sequencing, and all copy number changes were confirmed by microarrays that were performed concurrently with the WGS (Fig. 1a–c). No alternative pathogenic SNVs, indels or canonical SVs were identified, and the cxSVs were absent in our internal cohort of 9453 genomes, ClinVar or DECIPHER. The cxSVs in P1, P2 and P3 were therefore classified as pathogenic according to the ACMG guidelines [33].

Resolving the configuration of the cxSV in P4 was more challenging because the SV calls from short-read WGS were consistent with multiple possible configurations (Additional file 1: Figure S2). Importantly, in two of the possible configurations, there is an intact copy of *CDKL5* on the non-reference allele, in addition to the disrupted copy (Additional file 1: Figure S2A–B), whereas in others there is no intact copy of *CDKL5* (Additional file 1: Figure S2C–D). Therefore, resolving the configuration was essential for the interpretation of the pathogenicity of this variant. We attempted PCR amplification over the predicted new formed breakpoint junctions and could only amplify one supporting the disrupted *CDKL5*, due to repetitive sequence around the other breakpoints. Both duplications were confirmed by microarray.

In order to resolve the configuration, we performed long-read WGS of P4 using ONT. We obtained a median read length of 8136 bp (Additional file 1: Figure S3A), 56% of the genome was covered with a minimum coverage of 3x (Additional file 1: Figure S3B), and around 97% of the reads mapped to the human genome (GRCh37/hg19). All the breakpoints of the cxSV were covered by at least four reads. Coverage was insufficient to resolve the cxSV using long-read SV calling algorithms such as Sniffles [25] or NanoSV [27] (for which a minimum coverage of 10x is recommended). In lieu of this, we manually reviewed the split long reads across the cxSV junction breakpoints. Eight of the reads that covered the cxSV breakpoints were identified as inherited from the paternal chromosome, either by SNP phasing (Fig. 1d, J2, J3, J4 and J6) or by indirect phasing based on the assumption that breakpoint junctions occur on the same allele (Fig. 1d, J5). Therefore, ONT sequencing allowed us to identify two reads supporting the junction that was initially not possible to confirm by Sanger sequencing (J5) due to repetitive sequences. By phasing analysis, we were also able to identify three reads supporting an intact copy of *CDKL5* in the allele inherited from the father (Fig. 1d, J6), confirming that the cxSV harbours an intact copy of *CDKL5*. Two possible configurations remain (Additional file 1: Figure S2A–B), both of which have been proposed previously [12, 34]. These are indistinguishable by short-read sequencing technology because the breakpoint junctions are identical, or even by long reads unless all junctions are crossed in the same molecule. Only one of these possible configurations is represented in Fig. 1d and Table 1 for clarity.

We performed RNA expression analysis (Sanger sequencing of one informative SNP using cDNA) and demonstrated biparental allele expression of *CDKL5* in the child (Additional file 1: Figure S4). This further supports the presence of an intact copy of *CDKL5* on the paternal allele and suggests that regulation of *CDKL5* is probably not perturbed by the nearby cxSV. This variant was classified as VUS. The child is currently 1 year old and developmentally normal with no seizures, but remains under ongoing follow-up.

### Microhomology and repetitive elements occur in conjunction at cxSV breakpoint junctions

Mutational signatures around novel breakpoint junctions of SVs can yield insights into the mechanisms by which they were formed. Therefore, we analysed the sequences of all of the novel breakpoint junctions. It has previously been reported that DNA replication-based mechanisms such as microhomology-mediated break-induced replication (MMBIR) or fork stalling and template switching (FoSTeS) are likely to be the primary mechanism responsible for the formation of cxSVs [3, 4, 35–37]. Our data overall support this as there is microhomology of at least 3 bp in all of the eight novel breakpoint junctions in the four individuals (Additional file 1: Figure S5 and Additional file 2: Table S2). We also observe in P2 the insertion of two sequences of 5 and 48 bp in J1 and J3 junctions, and the insertion in P4 of a 100 bp *Alu* sequence in J2 junction. It has been previously suggested that *Alu* elements could facilitate template switching and annealing via homology between replication forks [37].

Additional evaluation of the breakpoint sequences with RepeatMasker also identified longer repetitive elements in all of the individuals (Table 2 and Additional file 1: Figure S5). In P1, we found that sequence flanking two of the breakpoints had high similarity to SINE sequences (ERVL-MaLRs), one with LINE sequences (L2) and one with DNA/hAT-Charlie (MER3) sequences (Table 1); in P2, we noted that sequence flanking three of the breakpoints had similarity to SINE sequences (*Alu* and MIR); in P3, sequences surrounding all the breakpoints presented high similarity to LINEs; and in P4, one of the breakpoints had similarity to SINE/Alu sequences.

**Table 2:** Repetitive elements associated with cxSV reference breakpoints in the four participants

Participant	Breakpoint^a^	Coordinate of breakpoint	Repetitive elements
P1	A3′-B5′	6:151443332	91% LTR/ERVL-MaLR
	B3′-C5′	6:154768570	32% DNA/hAT-Charlie
	C3′-D5′	6:154774047	75% LTR/ERVL-MaLR
	D3′-E5′	6:154778901	26% LINE/L2
	E3′-F5′	6:154778992	–
P2	A3′-B5′	1:244867200	70% SINE/Alu
	B3′-C5′	1:246064238	–
	C3′-D5′	1:246569871	45% SINE/MIR
	D3′-E5′	1:246816211	88% SINE/Alu
P3	A3′-B5′	9:80843698	82% LINE/L1 and 14% SINE/Alu
	B3′-C5′	9:80849462	59% LINE/L1
	C3′-D5′	9:80849760	63% LINE/L1
	D3′-E5′	9:80859678	86% LINE/L1
P4	A3′-B5′	X:17793009	–
	B3′-C5′	X:18074005	–
	C3′-D5′	X:18248955	41% SINE/Alu
	D3′-E5′	X:18532312	–

^a^Refers to genomic segments as shown in Fig. 1. Repetitive elements, identified using RepeatMasker, represent the percentage of repetitive sequence for a 300 bp region of reference sequence flanking the breakpoint, as specified element: class/family

A recent study showed that a high proportion of *Alu*-mediated SVs contain a hybrid *Alu* element in the derivative chromosome [34]. However, we do not observe these in our study. Studies have also shown that due to the error-prone nature of replication-based mechanisms of cxSV formation, de novo SNVs and indels can occur concomitantly to cxSVs [38]. Thus, we looked for de novo SNVs or indels in 1 Kb regions around each novel breakpoint junction in those individuals for which parental WGS data was available (P1 and P4), and there were none. For P2 and P3, we considered all rare SNVs and indels in those regions and did not identify any.

## Discussion

In the present study, we aimed to identify cxSVs relevant to Mendelian disease using short-read WGS, to resolve the precise variant configurations and to investigate possible mechanisms of cxSV formation. We have presented three individuals with pathogenic cxSVs and one with an interesting cxSV of unknown significance. We showed that precise resolution of variant configuration can be essential for interpreting pathogenicity and presented evidence of both DNA replication based and homologous recombination mechanisms of formation.

Here, we highlight the role of cxSVs as a cause of Mendelian disease. However, cxSVs are not typically considered in analysis pipelines, in part due to the technical and analytical challenges around identification and interpretation, and when there is an associated deletion or duplication detected by microarray further analysis is rarely performed. Therefore, when not included in analyses, the full scope of genome-wide structural variation is overlooked. In this study, 0.2% (3/1324) of Mendelian disease cases were caused by cxSV. This compares to approximately 5–20% of individuals with Mendelian disorders who have a clinically relevant canonical SV [18, 39, 40]. However, 0.2% is likely to be an underestimate because short-read WGS has limited power to resolve cxSVs and because our workflow was designed to maximise specificity.

There are several technologies available for the identification of cxSVs, including short-read WGS, long-read WGS, long insert WGS (liWGS) and microarrays, each with strengths and limitations. This study demonstrates the potential of short-read WGS to identify clinically relevant cxSVs. An advantage of this is that it is a more commonly used technology than some of the other options and does not necessitate using a different technology specifically to identify cxSVs. The main limitation is that at repetitive regions mapping and variant calling algorithms have lower sensitivity. This is particularly problematic given that cxSVs are more likely to occur in repetitive regions. These limitations may now be addressed by long-read sequencing technologies such as Nanopore, either in combination with another technology as in this study or as a first line approach. These have the advantage of reads of 10–100 Kb allowing for more accurate mapping particularly over repetitive regions and facilitating phasing [27]. Various other studies have already demonstrated the power of long-read WGS to detect SVs and cxSVs [25, 27, 41, 42]. However, the limitations are that coverage is lower and error rate is higher than short-read WGS. For these reasons, it is unlikely that, in our case, these variants would have been identified from long-read data alone without prior knowledge of the region of interest. A third possible method to identify complex rearrangements is liWGS, which has been successfully employed to detect cxSVs in other studies, and has the advantage over short-read WGS of improved mapping particularly over repetitive regions due to the large fragments, but has a lower resolution of ~ 5 Kb [11–13].

The segments of cxSVs that have copy number changes could in some cases be detected by microarray. These have the advantage of low cost, and that they are already the first-line test in many cases. However, they do not allow precise resolution of breakpoints and they would also miss inversions and those cxSVs where there is no CNV change. Importantly, a routinely pre-screening by microarray could potentially identify a high number of cxSVs. Actually, it has been reported that 7.6% of all rare duplications detected by microarray are part of a complex rearrangement [12]. However, it is likely that many ‘canonical CNVs’ detected by microarray are actually misclassified cxSVs, in part due to the impossibility for detecting inversions by microarray, since inversions are involved in 84.8% of cxSVs [11]. In this study, the copy number changes of all four cxSVs were confirmed by microarrays.

Our experience with P4, whose cxSV intersects *CDKL5*, demonstrates that understanding the precise configuration of a cxSV can be essential for interpreting the pathogenicity of the variant, especially if the gene of interest is disrupted by a duplication or inversion rather than a deletion. The impact of a deletion on the function of affected genes is generally assumed to be LOF. However, the consequence of a duplication can be uncertain and depends on precisely how the variant rearranges the gene, as well as gene-specific factors such as dosage sensitivity. Furthermore, duplications intersecting regulatory regions can result in a different phenotype from variants within the gene itself [43].

Investigating cxSVs in our cohort identified previously reported subclasses (delINVdup, delINVdel and dupINVdup in P2, P3 and P4, respectively), as well as a dupINVinvDEL in P1 [11]. One of the limitations of our study is that certain subclasses of cxSVs such as chromothripsis and those formed by nested rather than chained breakpoints would be excluded by our filtering and clustering method. Our method was designed specifically to identify possibly pathogenic variants in Mendelian disease, and thus was optimised to maximise specificity, at the cost of sensitivity and scalability. Therefore, while a detailed, sensitive, large-scale assessment of the role of cxSVs in Mendelian disease would be valuable, this is beyond the scope of the present study. Even when a putative cxSVs is identified, sometimes it is not straightforward to define them because the distinction between canonical SVs, cxSVs and chromoanagenesis can be unclear [35, 38]. It is therefore perhaps appropriate to consider types of human genomic variation as a continuum rather than discrete classes, progressing from SNVs (that typically cause the least disruption to the genome), through indels, canonical SVs and cxSVs to the highly disruptive chromoanagenesis and aneuploidies.

The high frequency of microhomology observed at the breakpoint junctions of the cxSVs in our study and the presence of inserted sequence in three of them is consistent with the hypothesis that replication-based mechanisms such as FoSTeS/MMBIR are primarily responsible for the formation of cxSVs [3, 4, 35–37]. However, we also find longer repetitive elements including *Alu* elements in the vicinity of breakpoint junctions in all of the cxSVs, consistent with other studies [44, 45]. The exact role of repetitive elements in SV/cxSV formation is currently unclear. Repetitive elements have classically been seen as signatures of recombination-based mechanisms such as non-allelic homologous recombination, which is a well-known mechanism of formation of recurrent SVs [4, 44]. This could suggest that recombination-based and replication-based mutational mechanisms might together mediate the formation of non-recurrent cxSVs. However, it is more likely that repetitive elements facilitate replication-based SV/cxSV formation by, for example, providing the requisite microhomology islands or increasing the susceptibility of the region to the formation of secondary DNA structures that can cause replication fork collapse [46, 47].

## Conclusions

Our work demonstrates that cxSVs contribute to rare Mendelian disorders, and provides insight into identifying and resolving both the conformation and the mechanism of formation of cxSVs by using short and long-read WGS. We demonstrate that understanding the precise configuration can be essential for interpreting the pathogenicity of cxSVs. We suggest that cxSVs should be included into research and clinical diagnosis and considered when screening SVs in the human genome. Further detailed characterisation of cxSVs in large-scale WGS studies will be essential for further unveiling the complex architecture of cxSVs and determining accurate population frequencies.

## Additional files

Additional file 1: **Figure S1.** cxSV analysis workflow. **Figure S2.** cxSV models for P4. **Figure S3.** Quality control results of the long-read WGS performed on P4. **Figure S4.** RNA gene expression of *CDKL5* from P4 and both parents. **Figure S5.** Proposed mechanisms of cxSV formation and breakpoint junction alignments. (PDF 1966 kb)

Additional file 2: **Table S1.** List of genes present in P1, P2, P3 and P4 cxSVs). **Table S2.** Complex SVs breakpoint details) (XLSX 32 kb)

## Abbreviations

CSS: Coffin-Siris syndrome
cxSV: Complex structural variant
EIEE: Early infantile epileptic encephalopathy
FoSTeS: Fork stalling and template switching
IRD: Inherited retinal disorder
liWGS: Long insert WGS
LOF: Loss-of-function
MMBIR: Microhomology-mediated break-induced replication
NDD: Neurological and developmental disorder
ONT: Oxford Nanopore Technologies
SNV: Single nucleotide variant
SV: Structural variant
WGS: Whole-genome sequencing
