# Supplementary material for: Complex structural variants in Mendelian disorders: identification and breakpoint resolution using short- and long-read genome sequencing
Source: Genome Med. 2018 Dec 7;10:95. doi: 10.1186/s13073-018-0606-6 (PMC6286558; doi:10.1186/s13073-018-0606-6)
Supplement: Supplementary file 1 — Figure S1. cxSV analysis workflow. Figure S2. cxSV models for P4. Figure S3. Quality control results of the long-read WGS performed on P4. Figure S4. RNA gene expression of CDKL5 from P4 and both parents. Figure S5. Proposed mechanisms of cxSV formation and breakpoint junction alignments. (PDF 1966 kb) [file 13073_2018_606_MOESM1_ESM.pdf]

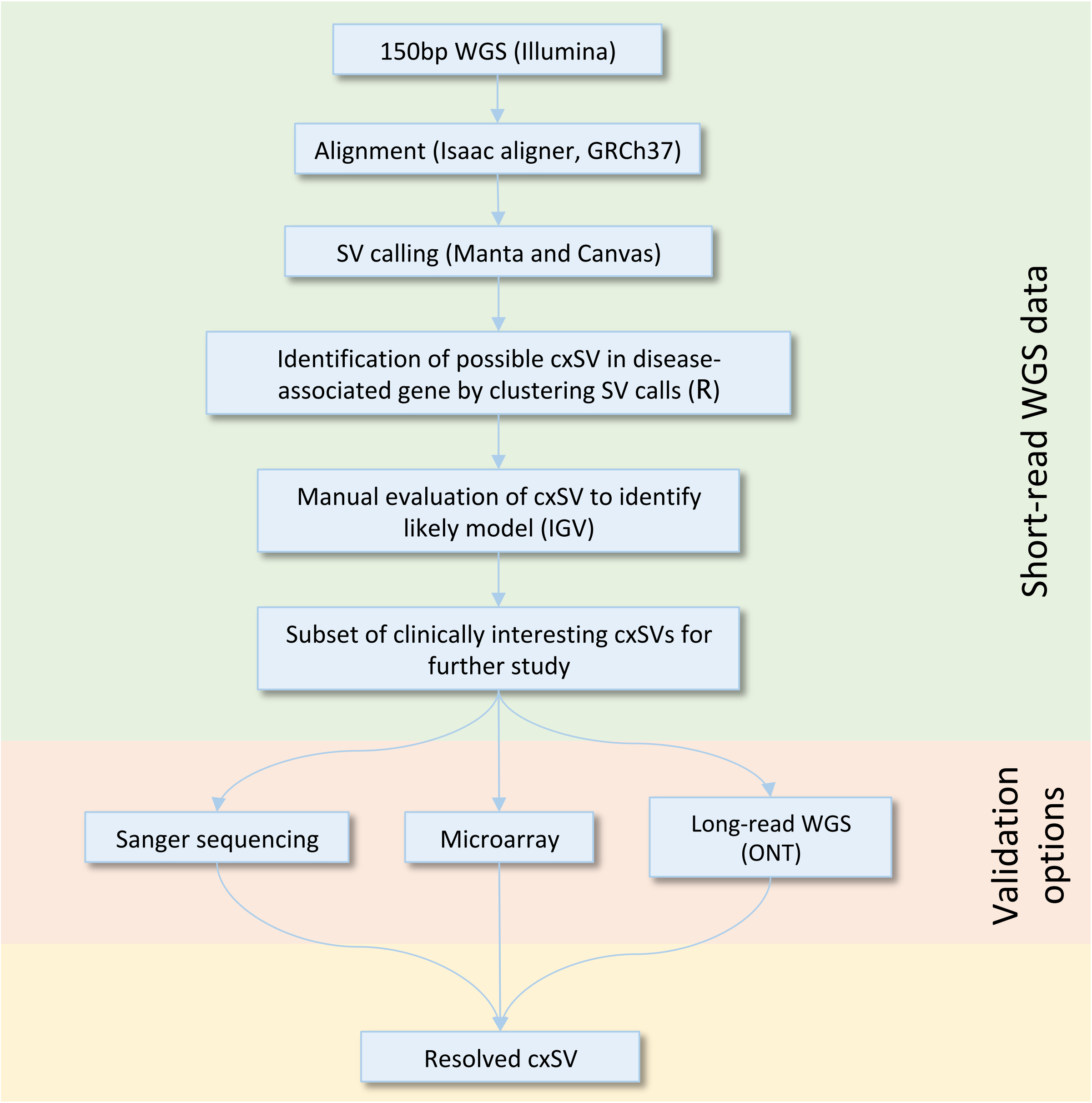

Figure S1. Complex structural variant analysis workflow. Schematic describing the general stages of complex structural variant analysis. Each variant was validated by at least two of the validation options. WGS = whole genome sequencing; SV = structural variant; cxSV = complex structural variant; IGV = Integrative Genomics Viewer; ONT = Oxford Nanopore Technologies.

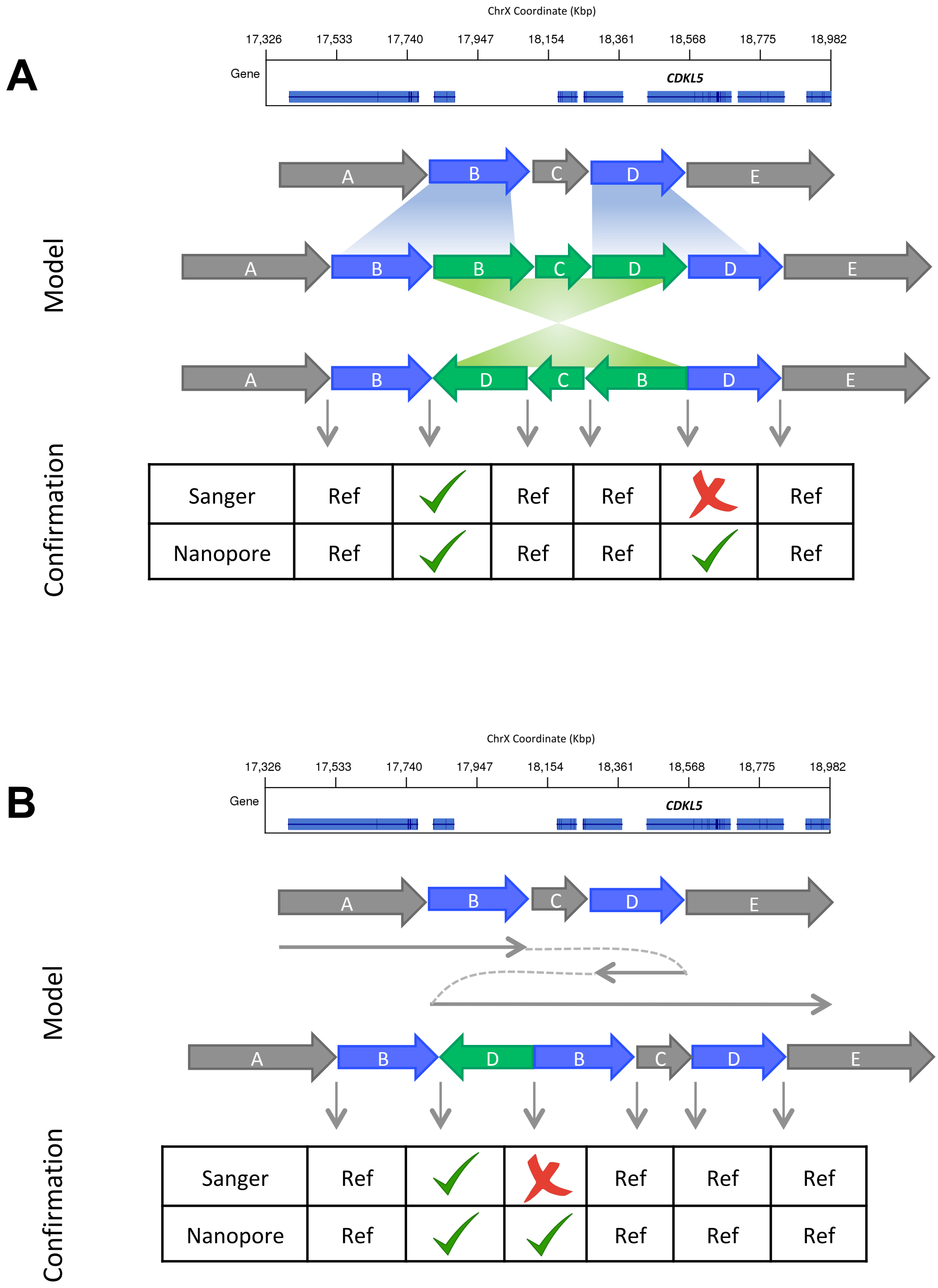

Sanger	Ref	✓	Ref	Ref	✗	Ref
Nanopore	Ref	✓	Ref	Ref	✓	Ref

Sanger	Ref	✓	✗	Ref	Ref	Ref
Nanopore	Ref	✓	✓	Ref	Ref	Ref

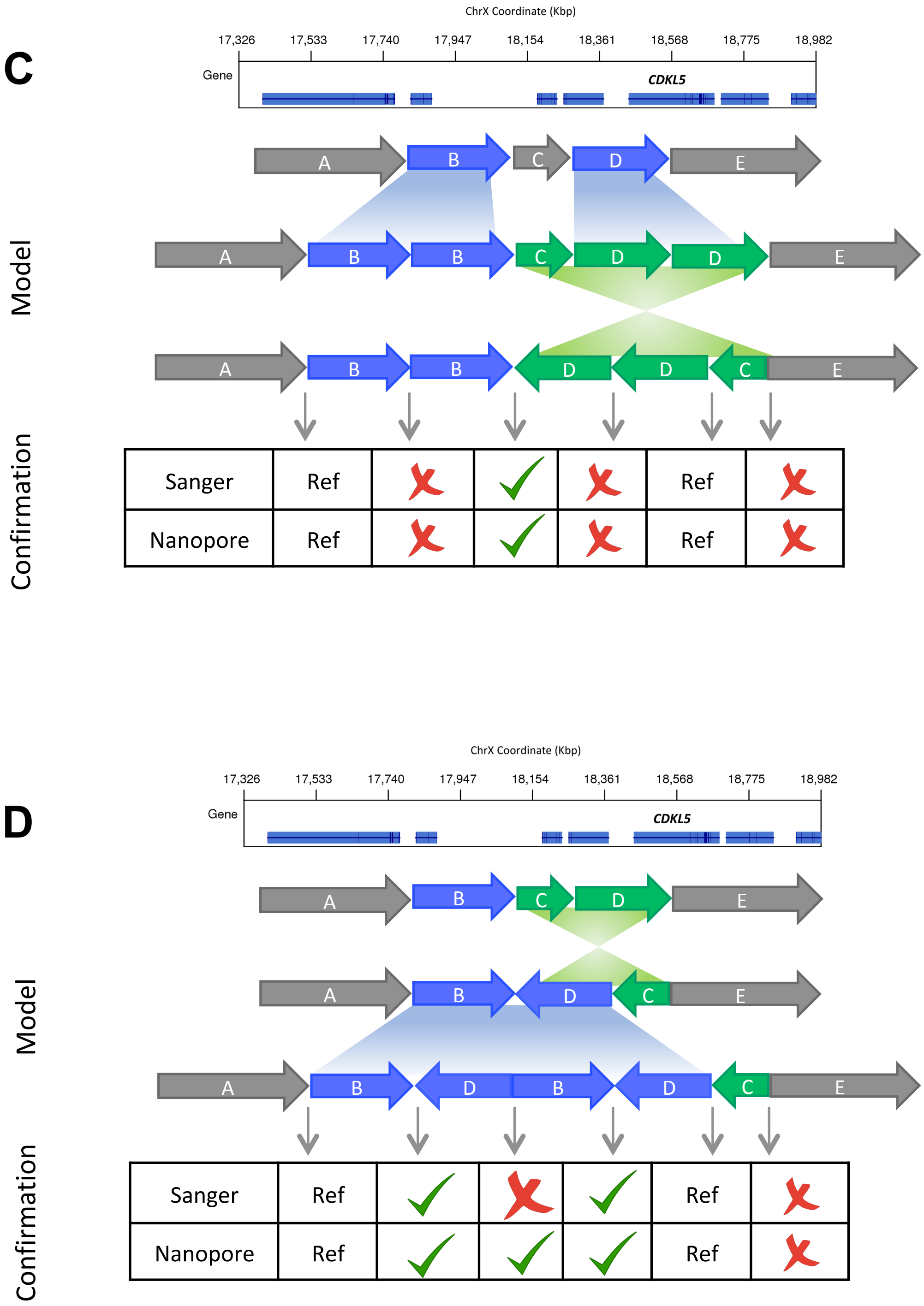

Figure S2. Complex structural variant models for Participant 4 (P4). Multiple possible models for the cxSV in P4 were considered, of which four are shown here. (**A**) Formed by two duplications and one inversion. Mechanism suggested by Brand et al. 2015[1]. Resulting junctions of this model were confirmed by Sanger and/or ONT sequencing. **(B)** Mechanism suggested by Gu et al. 2015[2], formed by one-step event. Resulting junctions of this model were confirmed by Sanger and/or ONT sequencing. (**C**) Rejected model. (**D**) Rejected model. Mechanism suggested by Gu et al. 2015[2]

A

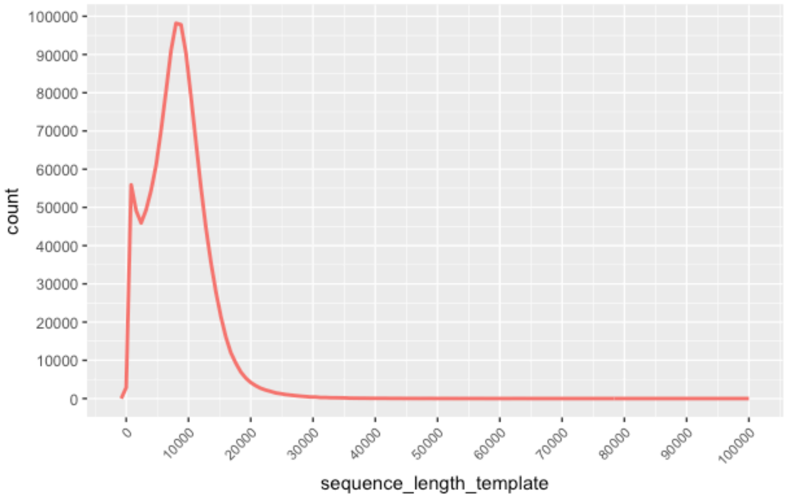

B

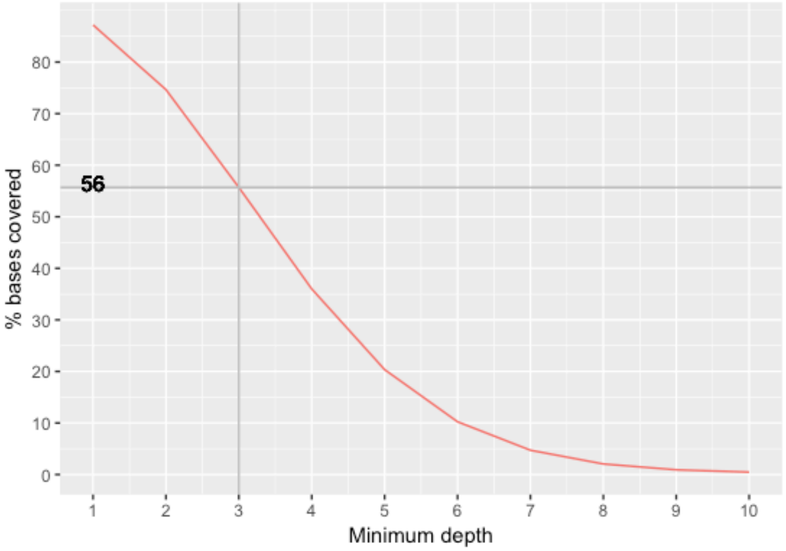

Figure S3. Quality control results of the long-read sequencing performed on P4. (**A**) Sequence length template distribution (**B**) Percentage of bases of the genome covered at a specific minimum coverage.

Informative SNP rs35478150
(GRCh37) Xg.18638082A>C

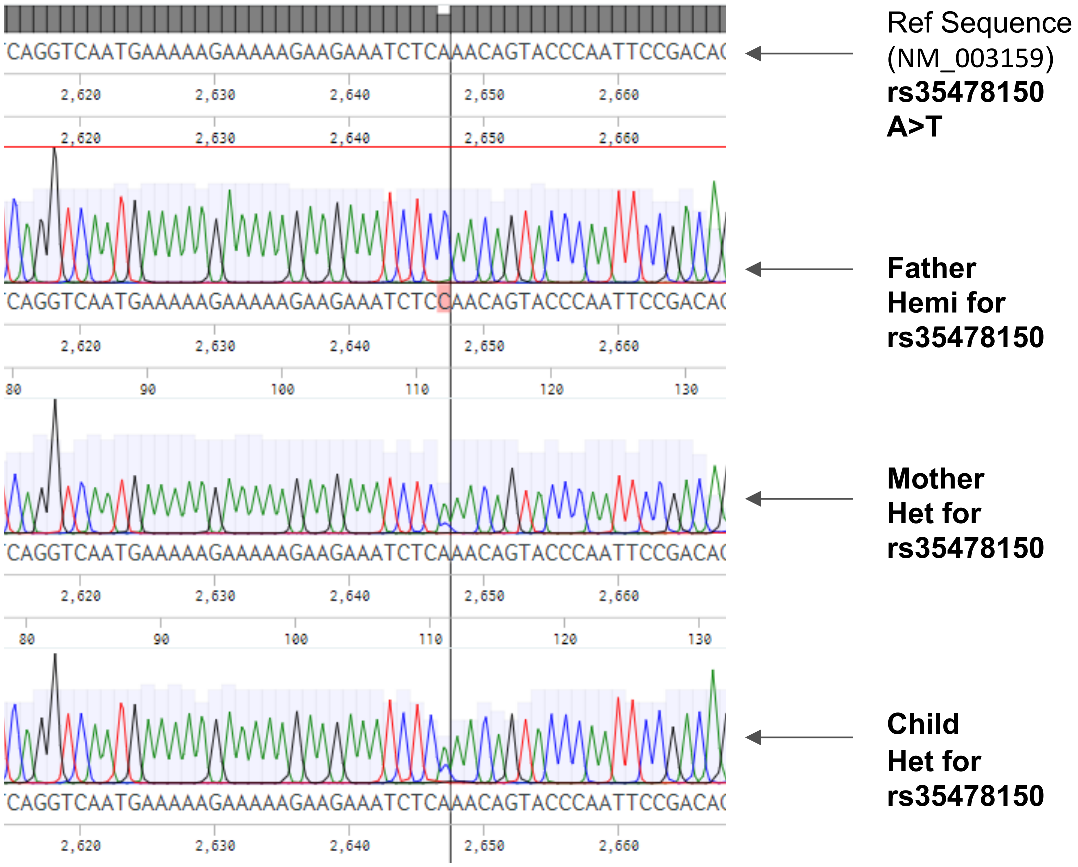

Figure S4. RNA gene expression of *CDKL5* from P4 and both parents. PCR amplification and Sanger sequencing of the informative SNP rs35478150 in the cDNA of *CDKL5* demonstrated that P4 expresses both alleles of *CDKL5* in blood cells.

A

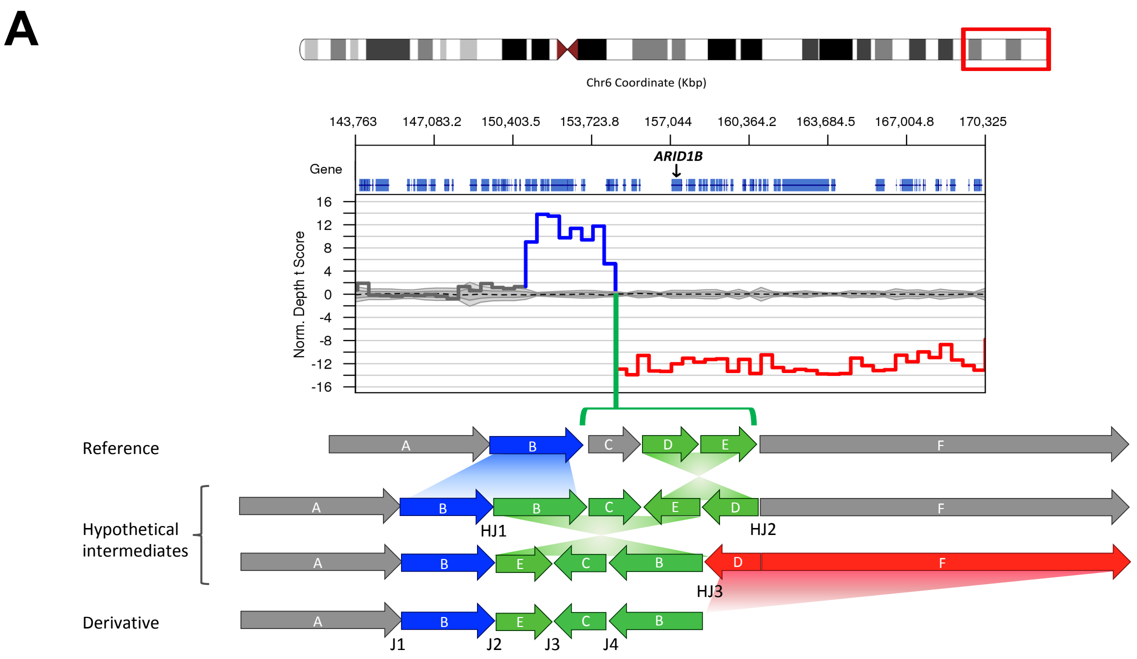

HJ1

```
6:151443283-151443342   GAGAGAGCCAGACAGAAGCTGTATCCTTGTTATGACCTAACCTGGAGGTCACCCAGCACT   LTR/ERVL-MaLR
HJ1               1-60  TATAATCGGTCTCAGGTACCCTCTACCCAGAGAGAGGAAGAAAAATTGGGACCCAGCACT
6:154768521-154768580   TATAATCGGTCTCAGGTACCCTCTACCCAGAGAGAGGAAGAAAAATTGGGTACAGATTCT

6:151443343-151443382   GTTTCCACCAAACTTCACTGACCAAGCTGATCACAAGCCT   LTR/ERVL-MaLR
HJ1             61-100  GTTTCCACCAAACTTCACTGACCAAGCTGATCACAAGCCT
6:154768581-154768620   AATTCAATCTGGGAAAGTGGTCCTGGTCTCATCATATAAT
```

J2

```
HJ1               1-60  TATAATCGGTCTCAGGTACCCTCTACCCAGAGAGAGGAAGAAAA-ATTGGGACCCAGCACT
P1                1-60  TATAATCGGTCTCAGGTACCCTCTACCCAGAGAGAGGAAGAAAA-ATTGGGTCCTCTATCA
6:154778852-154778911   ATAACCTAGAAAGTGGTGGCCATGCTCACTCCCTTCTTCCTCTTAATTCA-TCCTCTATCA   LINE/L2

HJ1             61-100  GTTTCCACCAAACTTCACTGACCAAGCTGATCACAAGCCT
P1              61-100  TTCCATCTGAGCAAAACCATCCTGTCCTTTACAGATCCCA
6:154778912-154778951   TTCCATCTGAGCAAAACCATCCTGTCCTTTACAGATCCCA
```

J3

```
6:154774097-154774040   CAAAGTTCCATAGACTGGGAGGCCGGAACAACAGAAATTTATCTCATCACC--ATTTTGG   LTR/ERVL-MaLR
P1                1-58  CAGATCCCAGTCCATTAGCTCAACACAGCATCTCAAGTCCTTTGGG--ACCCCATTTTGG
6:154778943-154779000   CAGATCCCAGTCCATTAGCTCAACACAGCATCTCAAGTCCTTTGGG--ACCCGTCCTCTT

6:154774039-154773998   AGGCTAGAAGTCTGAGATCGAGGTGTCAGCAGGGTAGGTTTC   LTR/ERVL-MaLR
P1              59-100  AGGCTAGAAGTCTGAGATCGAGGTGTCAGCAGGGTAGGTTTC
6:154779001-154779042   CCAAACCTCCCTGCAGTCCCATCATCCTGTAACTCACCCTAC
```

B

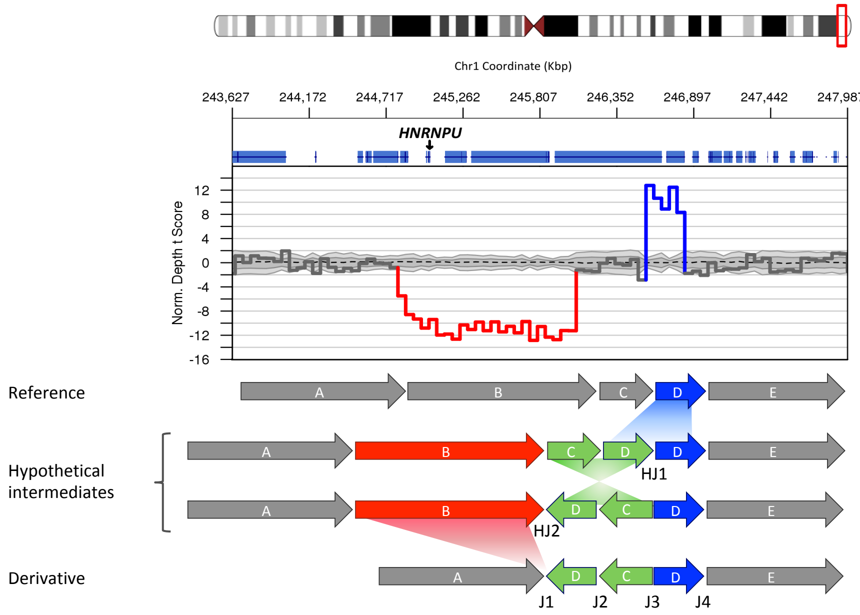

HJ1

```
1:246569822-246569881     TAAAAAGCTTGTCCTTGCCTTTTCGCTCTTCTTGTAAGTCTCTCTTCAGTGCACCGTGTC
HJ1                1-60   CGGGCGTGGTGGCGGGCGCCTGTAGTCCCAGCTACTCGGGAGGCTGAGGCGCACCGTGTC
1:246816162-246816221     CGGGCGTGGTGGCGGGCGCCTGTAGTCCCAGCTACTCGGGAGGCTGAGGCAGGAGAATGG   SINE/Alu

1:246569882-246569920     CATAGTGCTACGTGTTAGGCACTATTCAAAGCACATGACA   SINE/MIR
HJ1              61-100   CATAGTGCTACGTGTTAGGCACTATTCAAAGCACATGACA
1:246816222-246816261     CGTGAACCTGGGAGGCGGAGCTTGCAGTGAGCCGAGATTG   SINE/Alu
```

HJ2

```
1:246064189-246064244     TCCCAGGGCAGTTTCCCATGATTTCTGTCATTCTTGCCTGTGGCAAAAGG----CTAACA
HJ2                1-57   TCCCAGGGCAGTTTCCCATGATTTCTGTCATTCTTGCCTGTGGCAAAAGG---GCCTCAG
HJ1:-1             1-57   TGTCATGTGCTTTGAATAGTGCCTAACACGTAGCACTA--TGG-ACACGGTGCGCCTCAG

1:246064245-246064287     ATAAAGTAGATTGAATCTTCAGGAGCTATTAGAATATTCAACT
HJ2              58-100   CCTCCCGAGTAGCTGGGACTACAGGCGCCCGCCACCACGCCCG
HJ1:-1           58-100   CCTCCCGAGTAGCTGGGACTACAGGCGCCCGCCACCACGCCCG
```

J1

```
HJ2                1-54   TCCCAGGGCAGTTTCCCATGATTTCTGTCATTCTTGCCTGTGGCAAAAGG------GCCT
J1                 1-59   GGGGTTTCACCGTGTCAGCCAGGATGGTCTCGATCTCCTGACCTCATGATTCGCC-GCCT
1:244867151-244867210     GGGGTTTCACTGTGTTAGCCAGGATGGTCTCGATCTCCTGACCTCATGATCTGCCCGCCT   SINE/Alu

HJ2               55-94   CAGCCTCCCGAGTAGCTGGGACTACAGGCGCCCGCCACCA
J1                60-99   CAGCCTCCCAAAGTGCTGGGATTACAGGCGCCCGCCACCA
1:244867211-244867250     CGGCCTCCCAAAGTGCTGGGAATTACAGGTGTGAGCCACC   SINE/Alu
```

J3

```
HJ1                1-50   CGGGCGTGGTGGCGGGCGCCTGTAGTCCCAGCTACTCGGGAGGCTG--AGGC--------
J3                 1-54   AAGTTGAATATTCTAATAGCTCCTGAAGATTCAATCTACTTTATTGTTAGGGGT------
1:246064288-246064229     AAGTTGAATATTCTAATAGCTCCTGAAGATTCAATCTACTTTATTGTTAGCCTTTTGCCA

HJ1               51-66   --------------------------------------------GCACCGTGTCCATAGT
J3               55-114   CCCAGAAGAAGGCCCTCCTAGCCGACTACAACAGCAATCTCTAGGCACCGTGTCCATAGT
1:246064228-246064169     CAGGCAAGAATGACAGAAATCATGGGAAACTGCCCTGGGATTCCTCCCAGACCATGATCT

HJ1              67-106   GCTACGTGTTAGGCACTATTCAAAGCACATGACATGTACC
J3              115-154   GCTACGTGTTAGGCACTATTCAAAGCACATGACATGTACC
1:246064168-246064129     GGAATGTTAGCAAGGTAGCTTTCCACCAGCATCCTTGGTT
```

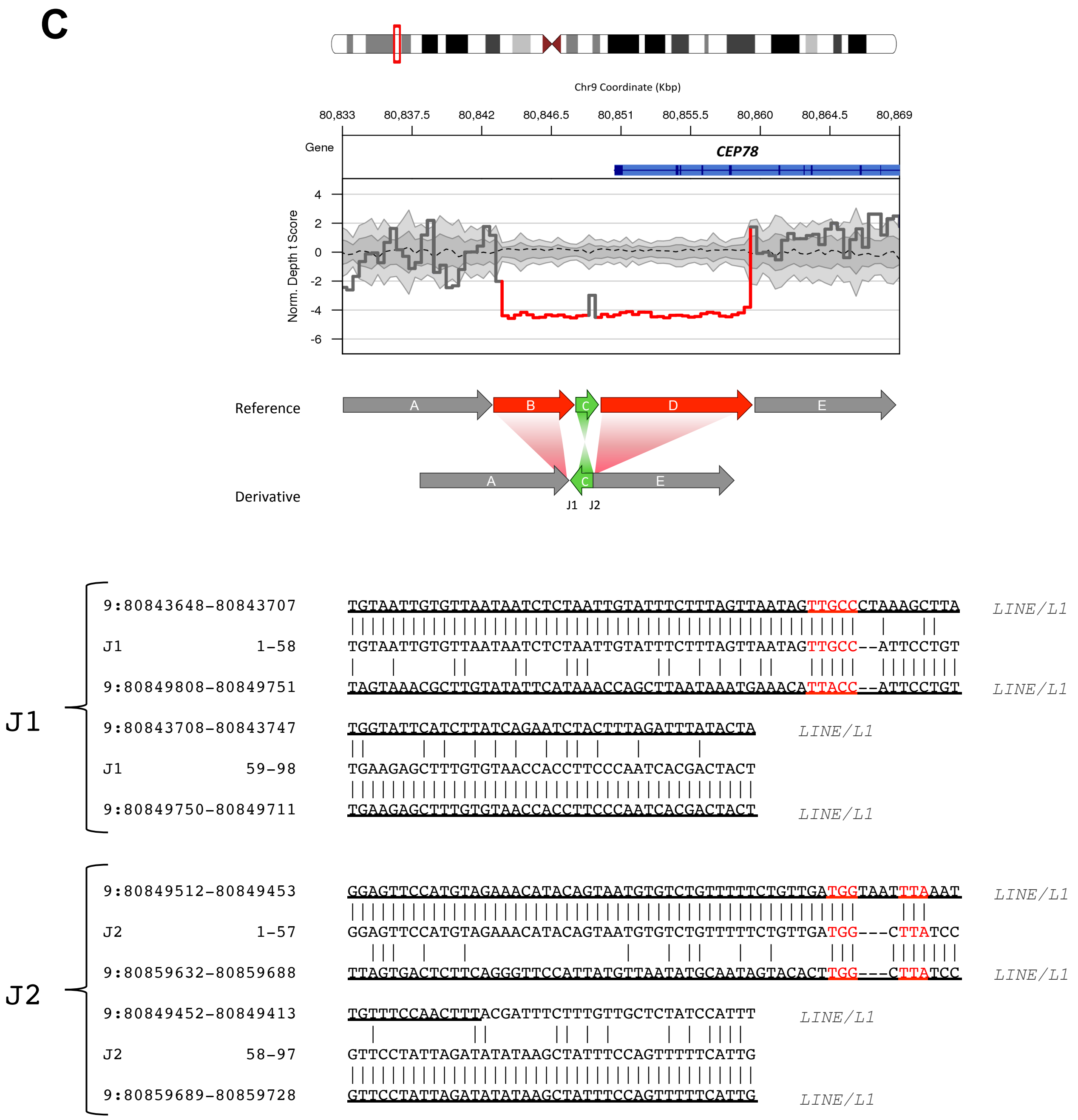

C
Chr9 Coordinate (Kbp)
80,833
80,837.5
80,842
80,846.5
80,851
80,855.5
80,860
80,864.5
80,869
Gene
CEP78
Norm. Depth t Score
4
2
0
-2
-4
-6
Reference
A
B
C
D
E
Derivative
A
C
E
J1
J2
J1
9:80843648-80843707 TGTAATTGTGTTAATAATCTCTAATTGTATTTCTTTAGTTAATAGTTGCCCTAAAGCTTA LINE/L1
J1 1-58 TGTAATTGTGTTAATAATCTCTAATTGTATTTCTTTAGTTAATAGTTGCC--ATTCCTGT
9:80849808-80849751 TAGTAAACGCTTGTATATTCATAAACCAGCTTAATAAATGAAACATTACC--ATTCCTGT LINE/L1
9:80843708-80843747 TGGTATTCATCTTATCAGAATCTACTTTAGATTTATACTA LINE/L1
J1 59-98 TGAAGAGCTTTGTGTAACCACCTTCCCAATCACGACTACT
9:80849750-80849711 TGAAGAGCTTTGTGTAACCACCTTCCCAATCACGACTACT LINE/L1
J2
9:80849512-80849453 GGAGTTCCATGTAGAAACATACAGTAATGTGTCTGTTTTTCTGTTGATGGTAATTTAAAT LINE/L1
J2 1-57 GGAGTTCCATGTAGAAACATACAGTAATGTGTCTGTTTTTCTGTTGATGG---CTTATCC
9:80859632-80859688 TTAGTGACTCTTCAGGGTTCCATTATGTTAATATGCAATAGTACACTTGG---CTTATCC LINE/L1
9:80849452-80849413 TGTTTCCAACTTTACGATTTCTTTGTTGCTCTATCCATTT LINE/L1
J2 58-97 GTTCCTATTAGATATATAAGCTATTTCCAGTTTTTCATTG
9:80859689-80859728 GTTCCTATTAGATATATAAGCTATTTCCAGTTTTTCATTG LINE/L1

D

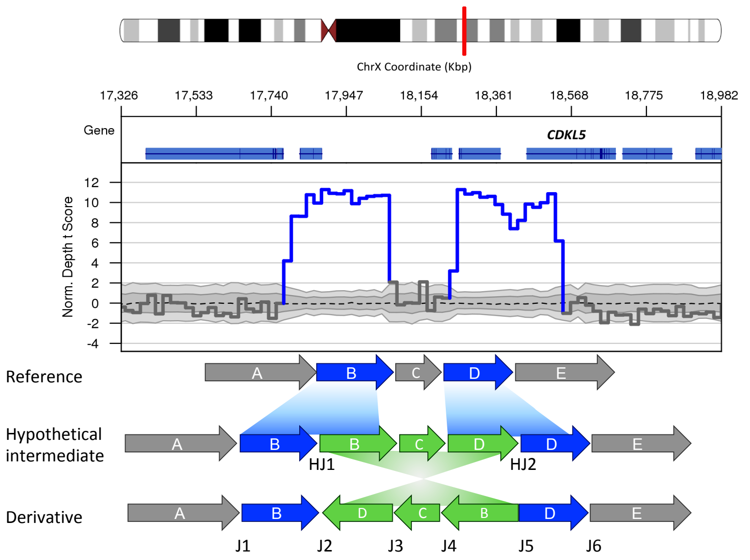

HJ1

```
X:17792960-17793019   ACATGATCTGCCTCTGCCCATGTCTTACTGTCTTTCTGACATTCGTACCCACACATGATG
HJ1            1-60   AGAAGACTAACTGAAAAAAGTTTGTTTTGTGTCTATATTTGGTGCCAGTTACACATGATG
X:18073956-18074015   AGAAGACTAACTGAAAAAAGTTTGTTTTGTGTCTATATTTGGTGCCAGTTTGTTGAGAAA

X:17793020-17793059   TGGCTCTGACCATCTCTTCCTGTCTTCCTTTCATTGGTAC
HJ1          61-100   TGGCTCTGACCATCTCTTCCTGTCTTCCTTTCATTGGTAC
X:18074016-18074055   ACCCTATCTAACTTGATGTTATTCAAGCATGCCCAACAGC
```

HJ2

```
X:18249005-18248946   CATGAGCCACCATGCCCAGCCTATGCCTTATTGTATTACACTCAATGCCAACACATGATG   SINE/Alu
HJ2            1-60   CATGAGCCACCATGCCCAGCCTATGCCTTATTGTATTACACTCAATGCCACTGGATCAGC
X:18532362-18532303   TGAAATTGAGCAGCATTAATTTCAGTAAATTAGGAAGTATGATCTTTGGTCTGGATCAGC

X:18248945-18248906   TGGCTCTGACCATCTCTTCCTGTCTTCCTTTCATTGGTAC
HJ2          61-100   CCAACATCATATTCTATGCACAAAAGCAAAATACACCTAT
X:18532302-18532263   CCAACATCATATTCTATGCACAAAAGCAAAATACACCTAT
```

J2

```
HJ1            1-60   AGAAGACTAACTGAAAAAAGTTTGTTTT-GTGTCTATATTTGGTGCCAGTTACACATGATG
J2             1-60   AGAAGACTAACTGAAAAAAGTTTGTTTT-GTGTCTATATTTGGTGCCAGTTGCACAATCAC   SINE/Alu
HJ2            3-50   GAGCCACCATGCCCAGCCTATGCCTTATTGTATTACACTCAA-TGCCA-------------

HJ1          61-120   TGGCTCTGACCATCTCTTCCTGTCTTCCTTTCATTGGTACTCACACAGGATCTGCCCCCC
J2           61-115   AG-CTCACTGCAGC-CTTGA---CTTCCCAGGCTCAAGTGATGCTCCCACCTCAGCCTCC   SINE/Alu
HJ2           50-50   ------------------------------------------------------------

HJ1         121-180   CACCCCCCTTCTCTTCTGTCTTTCTGTGCTCCATCCTCACACCTCTTCTGCTCCTGTCCG
J2          116-175   TGAGTAGCTGGGACTATACACATGAACCACCACACCTGGATCAGCCCAACATCATATTCT   SINE/Alu
HJ2           51-75   -----------------------------------CTGGATCAGCCCAACATCATATTCT

HJ1         181-200   TGTCTTTCTGTTTTAGTAGG
J2          176-175   ATGCACAAAAGCAAAATACA
HJ2           76-95   ATGCACAAAAGCAAAATACA
```

J5

```
HJ1          100-40   GTACCAATGAAAGGAAGACAGGAAGAGATGGTCAGAGCCACATCATGTGTAACTGGCACC
J5             1-60   GTACCAATGAAAGGAAGACAGGAAGAGATGGTCAGAGCCACATCATGTGTTGGCATTGAG
HJ2          100-43   GGTGTATTTTGCTTTTGTGCATAGAATATGATGTTGGGCTGATCCAGTG---GCATTGAG

HJ1            39-1   AAATATAGACACAAAACAAACTTTTTCAGTTAGTCTTCT
J5           61-100   TGTAATACAATAAGGCATAGGCTGGGCATGGTGGCTCATG
HJ2            42-3   TGTAATACAATAAGGCATAGGCTGGGCATGGTGGCTCATG
```

Figure S5. Proposed mechanisms of cxSV formation and breakpoint junction alignments for P1 (A), P2 (B), P3 (C) and P4 (D). Breakpoint junction sequence is aligned to the proximal and distal genomic reference sequence or sequence of breakpoint junction in hypothetical intermediate, as shown. Alignment is only shown for novel breakpoint junctions in the derivative chromosome, and the precursor hypothetical intermediate, if any. Microhomology at the breakpoint is indicated in red. Sequence in blue indicates inserted sequences at the breakpoint junction. Underline indicates repetitive elements in the reference, specified in *Italic*. J=Junction; HJ=Hypothetical junction.

References

1. Brand H, Collins RL, Hanscom C, Rosenfeld JA, Pillalamarri V, Stone MR et al **Paired-Duplication Signatures Mark Cryptic Inversions and Other Complex Structural Variation.** *Am J Hum Genet 2015, 97(1)170-176.*

2. Gu S, Yuan B, Campbell IM, Beck CR, Carvalho CMB, Nagamani SCS et al **Alu-mediated diverse and complex pathogenic copy-number variants within human chromosome 17 at p13.3**. *Hum Mol Genet. 2015 Jul 15; 24(14) 4061–4077.*
